# Integration of single-cell sequencing with machine learning and Mendelian randomization analysis identifies the NAP1L1 gene as a predictive biomarker for Alzheimer's disease

**DOI:** 10.3389/fnagi.2024.1406160

**Published:** 2024-06-26

**Authors:** Runming Chen, Yujun Xie, Ze Chang, Wenyue Hu, Zhenyun Han

**Affiliations:** ^1^Department of Neurology, Beijing University of Chinese Medicine Shenzhen Hospital (Longgang), Shenzhen, China; ^2^Dongzhimen Hospital, Beijing University of Chinese Medicine, Beijing, China; ^3^Xiyuan Hospital, China Academy of Traditional Chinese Medicine, Beijing, China; ^4^Dongfang Hospital, Beijing University of Chinese Medicine, Beijing, China

**Keywords:** single-cell sequencing, machine learning, Mendelian randomization analysis, NAP1L1 gene, biomarker, Alzheimer's disease

## Abstract

**Background:**

The most effective approach to managing Alzheimer's disease (AD) lies in identifying reliable biomarkers for AD to forecast the disease in advance, followed by timely early intervention for patients.

**Methods:**

Transcriptomic data on peripheral blood mononuclear cells (PBMCs) from patients with AD and the control group were collected, and preliminary data processing was completed using standardized analytical methods. PBMCs were initially segmented into distinct subpopulations, and the divisions were progressively refined until the most significantly altered cell populations were identified. A combination of high-dimensional weighted gene co-expression analysis (hdWGCNA), cellular communication, pseudotime analysis, and single-cell regulatory network inference and clustering (SCENIC) analysis was used to conduct single-cell transcriptomics analysis and identify key gene modules from them. Genes were screened using machine learning (ML) in the key gene modules, and internal and external dataset validations were performed using multiple ML methods to test predictive performance. Finally, bidirectional Mendelian randomization (MR) analysis, regional linkage analysis, and the Steiger test were employed to analyze the key gene.

**Result:**

A significant decrease in non-classical monocytes was detected in PMBC of AD patients. Subsequent analyses revealed the inherent connection of non-classical monocytes to AD, and the NAP1L1 gene identified within its gene module appeared to exhibit some association with AD as well.

**Conclusion:**

The NAP1L1 gene is a potential predictive biomarker for AD.

## 1 Introduction

Alzheimer's disease (AD) is a neurodegenerative condition characterized by progressive memory impairment, cognitive decline, and behavioral abnormalities (Scheltens et al., 2021). The total cost of treating AD was estimated at $305 billion in 2020, and it is expected to exceed $1 trillion by 2050. Additionally, the prevalence of AD is rising every year, with approximately 50 million people worldwide currently living with dementia. This number is projected to triple by 2050 (Dubois et al., 2016; Wong, 2020). Unfortunately, there is still no treatment available to provide a complete cure for AD. AD is a progressive condition that starts with mild memory problems, gradually leading to cognitive impairment and difficulty performing daily activities in about a decade. Administering neuroprotective drugs early—before mild symptoms appear—is a key strategy for treating AD. Therefore, identifying pre-AD stages and finding biomarkers to detect the pre-AD condition are especially important. Current research on AD biomarkers mainly uses techniques such as positron emission tomography (PET), cerebrospinal fluid Aβ1-42, and magnetic resonance imaging (MRI). However, none of these methods has been completely effective in identifying individuals at risk of developing early or full-blown AD (Fiandaca et al., 2014).

The advent of single-cell sequencing and transcriptomics has led to the introduction of new methods for identifying biomarkers for AD. Wang and Wang (2020) identified UBB, UBA52, SRC, MMP9, VWF, GP6, and PF4 as potential key genes for predicting AD. Yu et al. (2023) found that the lysosome-related gene ATP6V1E1 demonstrated a strong predictive performance for AD. These studies found potential biomarkers for AD but did not explore the causal relationship between AD and these biomarkers. In this study, Mendelian randomization (MR) analysis was introduced to establish a causal link between genes and AD. MR analysis was utilized to assess the causality of observed associations between modifiable exposures or risk factors and clinically relevant outcomes (Sekula et al., 2016). This study also revealed that monocytes were initially found to decline most significantly in the peripheral blood mononuclear cells (PMBCs) of AD patients. An imbalance between the production of Aβ and its clearance is thought to be an important cause of AD production. It has been shown that bone marrow- or blood-derived monocytes bind to Aβ deposits and are more effective phagocytes of Aβ than resident microglia (Zuroff et al., 2017).

Inspired by previous study, we hypothesized that a decrease in certain components of monocytes may impair Aβ clearance, contributing to the development of AD. Subsequently, we observed the greatest decrease in non-classical monocytes in AD patients, prompting us to perform single-cell transcriptome analyses on these monocytes, such as single-cell regulatory network inference and clustering (SCENIC) analysis. Eventually, we discovered the NAP1L1 gene. NAP1L1 is a member of the nucleosome assembly protein (NAP) family, ubiquitously expressed and involved in DNA replication, cell adhesion, migration, and proliferation (Yan et al., 2016; Dominguez et al., 2021; Peng et al., 2023). NAP1L1 has primarily been studied as a potential biomarker for tumors, but recent findings suggest it may have novel value in other areas as well (Nagashio et al., 2020; Shen et al., 2022). The study found that rats with a knockout of Nap1L1 exhibited slower proliferation of neural progenitor cells (NPCs) and premature differentiation of neurons during cortical development. Similarly, AD-induced pluripotent stem cell (AD-iPSC)-derived neural progenitor cells (AD-NPCs) showed premature neuronal differentiation, resulting in decreased proliferation and increased apoptosis, along with elevated levels of Aβ42 and phosphorylated tau (Vanova et al., 2023). This finding suggests that premature neuronal differentiation may be a contributing factor to AD. Therefore, NAP1L1 has significant potential as an AD biomarker.

**Figure 1:**
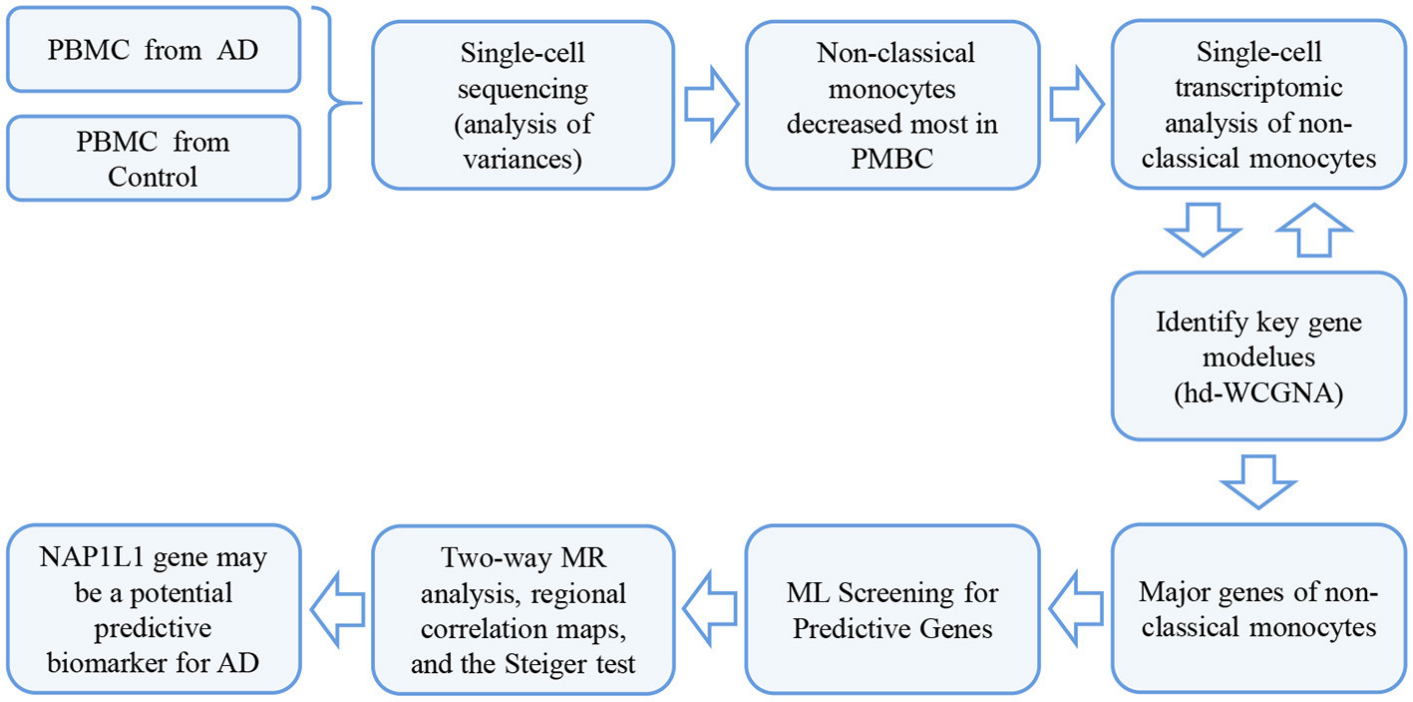
The workflow of the study.

In this study, we used high-dimensional weighted gene co-expression network analysis (hdWGCNA) to identify key genes, followed by machine learning (ML) to screen for predictive genes. Subsequently, we employed MR correlation analysis to explore the causal relationship between these genes and AD. Finally, we identified the NAP1L1 gene as a potential biomarker for AD as shown in the flow chart in Figure 1.

## 2 Materials and methods

### 2.1 Single-cell sequencing data download and processing

The Gene Expression Omnibus (GEO) database (https://www.ncbi.nlm.nih.gov/geo/) was searched using the keywords “Alzheimer's disease”, “peripheral blood”, and “human”. Subsequently, the dataset GSE226602 was retrieved, comprising AD (*n* = 28) and the control groups (*n* = 22). The R package “Seurat” was used for subpopulation annotation of single cells. To ensure data quality, we conducted quality control, data normalization, and other pre-processing steps to eliminate low-quality cells and alleviate inter-sample variability. During the downscaling and clustering process, we initially selected the genes with the highest variability for principal component analysis. Furthermore, we utilized the Harmony algorithm and employed the uniform manifold approximation and projection (UMAP) technique to visualize the data in two dimensions, showing diverse cell subpopulations in an unsupervised manner. In addition to manual annotation, we investigated automated annotation using the SingleR package and presented the results through DimPlot.

### 2.2 Training and validation of set data for ML downloads

GSE140829 and GSE97760 were retrieved from the GEO database using the GEO query R package (version 4.0.2). We designated GSE140829 containing peripheral blood samples from AD (*n* = 204) and the control groups (*n* = 249) as the training set. GSE97760 (Naughton et al., 2015) containing peripheral blood samples from AD (*n* = 9) and the control groups (*n* = 10) was designated as the validation set.

### 2.3 Pseudotime analysis and cellular communication analysis

For datasets involving time series or developmental processes, we employed the Monocle package to reconstruct cell track and utilized the Cellchat package to investigate cellular communication and regulatory dynamics.

### 2.4 SCENIC analysis

SCENIC analysis was performed using pySCENIC (v0.10.0) from the hg19-tss-centered-10 kb-10species database (https://github.com/aertslab/pySCENIC).

### 2.5 hdWGCNA networking

hdWGCNA was established using the “hd-WGCNA” R package, a widely utilized method for identifying potential biomarkers of interest.

### 2.6 Enrichment analysis

The hub genes obtained from hdWGCNA were selected for enrichment analysis of the top 50 genes of the key modules. Functional enrichment analysis of the key module hub genes was performed using the Metascape website (https://metascape.org/gp/index.html).

### 2.7 LASSO regression and logistic regression analysis

LASSO regression analysis was conducted using the “glmnet” R package, while logistic regression analysis was conducted using the “glm” R package. Key genes obtained from “hdWGCNA” were selected and integrated with GSE140829. The initial screening of the gene was performed through LASSO regression and logistic regression analysis to identify genes suitable for use as the training set in ML.

### 2.8 Predictive model construction based on multiple ML algorithms

The R package “mlr3” was applied to build ML models, including k-nearest neighbor algorithm (kknn), linear discriminant analysis (lda), naive_bayes, logistic regression (log_reg), random forest (ranger), support vector machine (svm), and recursive partitioning with regression trees (rpart). The ROC curve analysis was performed using the “pROC” R package and visualized using the “ggplot2” R package. The ROC curve analysis was used to validate the diagnostic value of these models in GSE140829, using data GSE97760 as an external validation set.

### 2.9 Bidirectional MR analysis

The genes predicted by ML were used to find expression quantitative trait loci (eQTL) matching the genes available on the genome-wide association study (GWAS) website (http://gwas.mrcieu.ac.uk/datasets). MR analysis was performed using the TwoSampleMR software package. The eQTL data for gene expression were subsequently processed using the vcfR package using reverse Mendelian correlation tool variables.

### 2.10 Regional correlation maps and the Steiger test

Genotype data and associated data were examined to extract eQTL information relevant to the target genes. Subsequently, eQTLs located within specified regions were selected and formatted for mapping the regional associations. The mapping process employed the locus-comparer software package to visualize the association information between eQTLs and GWAS, providing an intuitive graphical representation for subsequent analyses. Finally, single nucleotide polymorphism (SNP) with the most significant combinations for each trait and their corresponding PMID were filtered using the Steiger filter test and summarized in a comprehensive results table.

### 2.11 GWAS summary statistics for AD and NAP1L1 eQTL

Our GWAS summary statistics for AD were sourced from the dataset ebi-a-GCST90027158 (Bellenguez et al., 2022), including individuals (*n*_AD_ = 111,326, *n*_Control_ 677663) of European ancestry. The majority of the patients are aged over 60 years and represent both sexes. Furthermore, eQTL data from GWAS (OpenGWAS ID: eqtl-a-ESG00000187109) include whole blood NAP1L1 expression data sourced from Europe, with 17,270 SNPs collected from 9,188 samples.

## 3 Results

### 3.1 Single-cell analysis of the transcriptome of the AD and control groups

**Figure 2:**
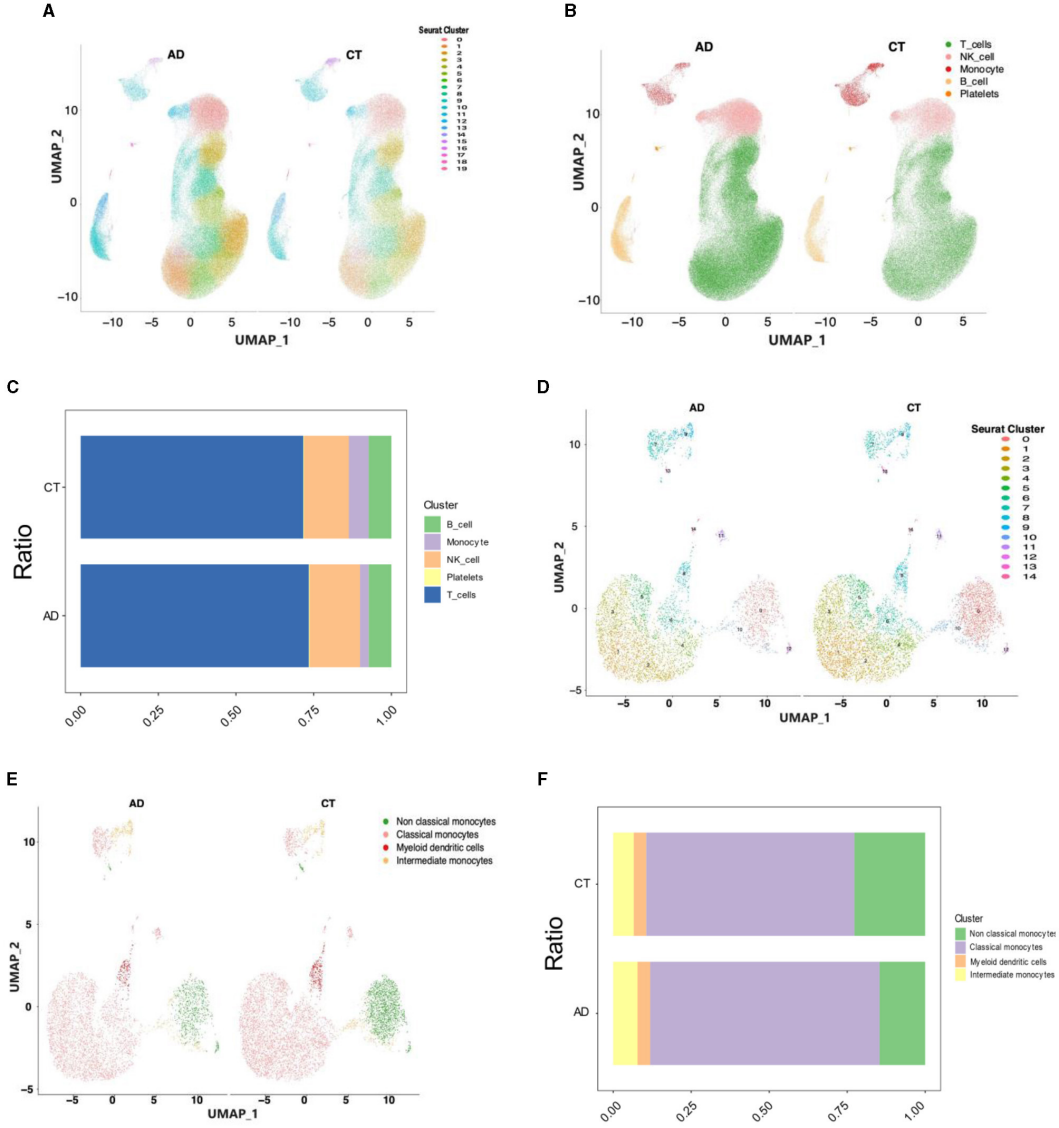
Single-cell analysis of the transcriptome of the AD and control groups. **(A)** The classification of cells into 20 subpopulations after the cluster analysis. **(B)** The classification of cells into five cell types after annotation using SingleR. **(C)** The ratios of five cell types. **(D)** The classification of monocytes into 15 subpopulations after the cluster analysis. **(E)** The classification of monocytes into four types after annotation using SingleR. **(F)** The ratios of four monocyte types.

In this study, PBMC samples from the AD (*n* = 28) and control groups (*n* = 22) from the dataset GSE226602 scRNA-seq were selected for analysis. Batch effects across samples were mitigated using the Harmony method to integrate and standardize the samples, followed by normalization, downscaling, and clustering. All cells were classified into 20 subpopulations using FindNeighbors and FindClusters functions of the Seurat software package, following quality control and clustering analyses of the data (Figure 2A). Using the SingleR software package, all cells were annotated into five cell types, namely, T cells, NK cells, monocytes, B cells, and platelets (Figure 2B). Subsequently, the ratios of five cell types are presented in Figure 2C. Notably, monocytes exhibited the most significant reduction and were consequently selected for subsequent analyses. Segmentation was continued based on monocytes, which were further segmented into non-classical monocytes, classical monocytes, myeloid dendritic cells, and intermediate monocytes. The distribution of monocytes was visualized utilizing the UMAP algorithm and DimPlot function and subsequently segmented based on cell type (Figures 2D, E). Finally, the ratios of four monocyte types are presented in Figure 2F. It was found that non-classical monocytes were the most significantly different among monocytes between the AD and control groups, and therefore non-classical monocytes were selected for subsequent analyses.

### 3.2 Single-cell transcriptomic analysis of non-classical monocytes

**Figure 3:**
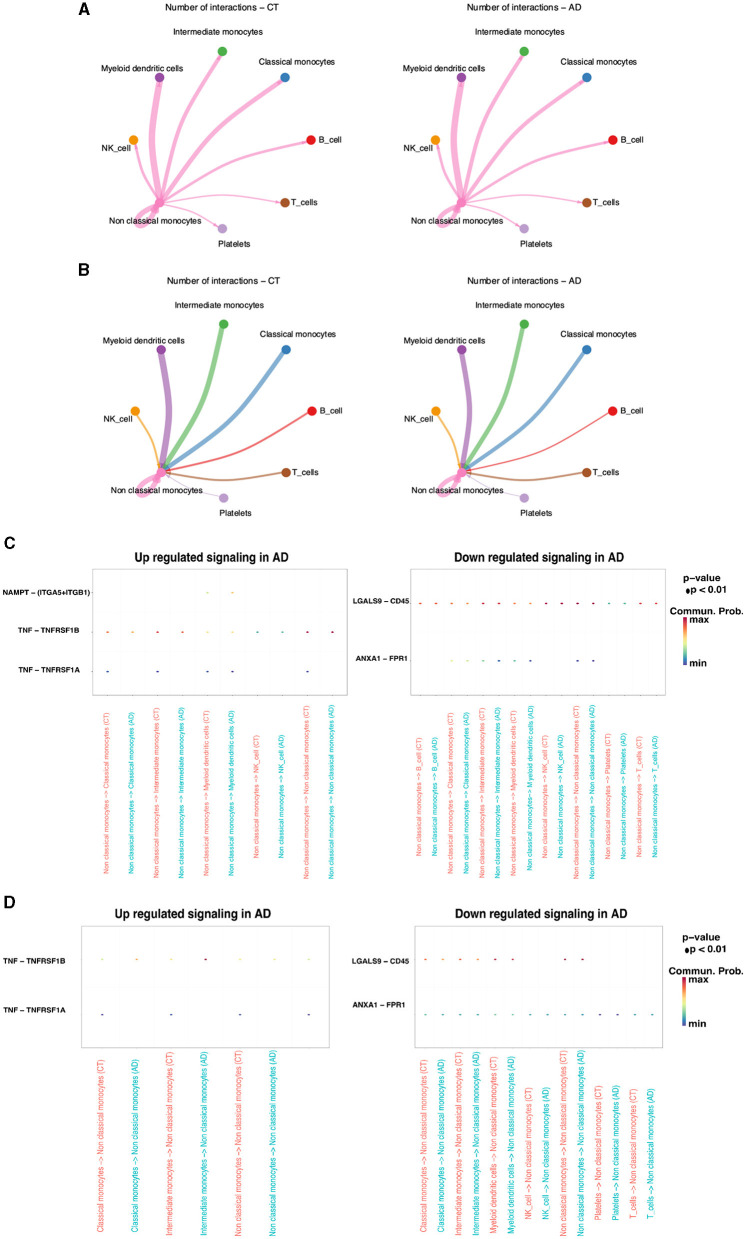
Cellular interactions between non-classical monocytes and other cells in the AD and control groups. **(A)** non-classical monocytes → other monocytes. **(B)** other monocytes → non-classical monocytes. Upregulated and downregulated signaling ligand-receptor pairs. **(C)** non-classical monocytes → other monocytes. **(D)** Other monocytes → non-classical monocytes.

Non-classical monocytes, which exhibited the most substantial decline in AD compared to the control group, were the focus of further single-cell transcriptomic analyses. To transition from the control group to an AD state, there must have been changes at the cellular level, including changes in intercellular communication. The aim was to comprehend the communication between non-classical monocytes and other monocytes. Initially, CellChat was used to infer the number of interactions between non-classical monocytes and other monocytes in both the AD and control groups. The results revealed that non-classical monocytes in AD did not exhibit interactions with other monocytes to the same extent as the control group (Figures 3A, B). To gain deeper insights into this cellular communication discrepancy, upregulated and downregulated signaling ligand–receptor pairs were identified through differential gene expression analysis. Subsequently, signaling differences were assessed based on the fold change of the ligand from the sending cell to the receptor in the receiving cell. The results indicated that NAMPT-(ITGA5-ITGB1), TNF-TNFRSF1B, and TNF-TNFRSF1A were signaling pathways upregulated in AD, while LGALS9-CD45 and ANXA1-FPR1 were signaling pathways downregulated in AD. The most prominent disparities in cellular communication between non-classical monocytes and other monocytes in both AD and control groups were observed in the TNF-TNFRSF1A signaling pathway (Figures 3C, D).

**Figure 4:**
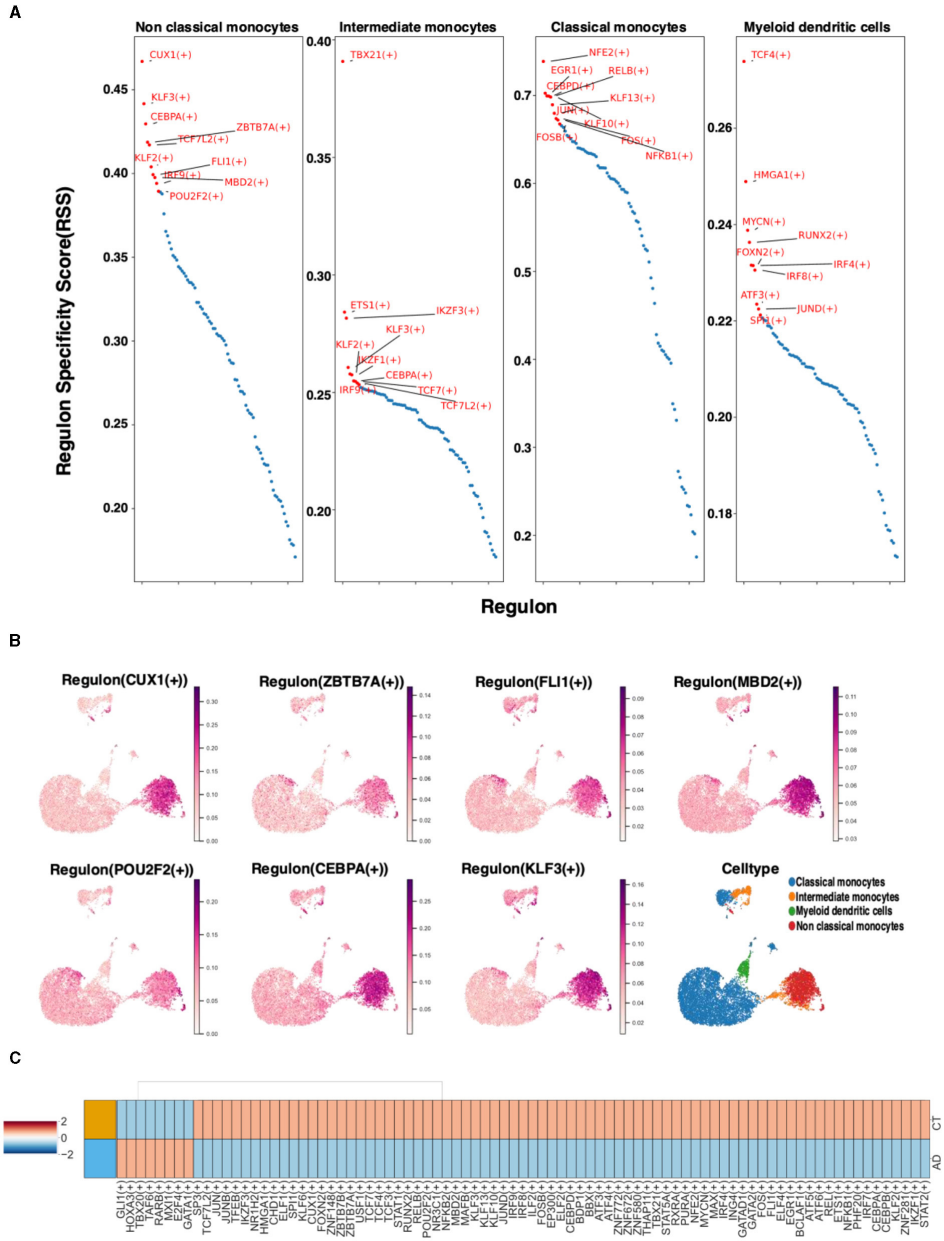
The case of non-classical monocyte transcription factors. **(A)** The RSS analysis of the top 10 specific regulators in different monocyte types. **(B)** Visualization of key transcription factors in non-classical monocytes. **(C)** Differences in the expression of the AD and control groups transcription factors in non-classical monocytes.

To comprehend the distinctions in single-cell transcriptional regulators between non-classical monocytes and other monocytes, the top 10 specific regulators in different monocytes were analyzed using the regulator specificity score (RSS). SCENIC analysis revealed that the top seven transcription factors in non-classical monocytes were CUX1, ZBTB7A, FL1, MBD2, POU2F2, CEBPA, and KLF3 (Figure 4A). Further examination of the distribution of these transcription factors in monocytes demonstrated that they were most prevalent in non-classical monocytes, which was consistent with previous findings (Figure 4B). To explore the potential association with AD, the top 100 transcription factors based on the RSS score were selected from non-classical monocytes. The differential expression of these transcription factors in the AD and control groups was observed. Notably, these transcription factors exhibited both upregulation and downregulation in both the AD and control groups (Figure 4C).

**Figure 5:**
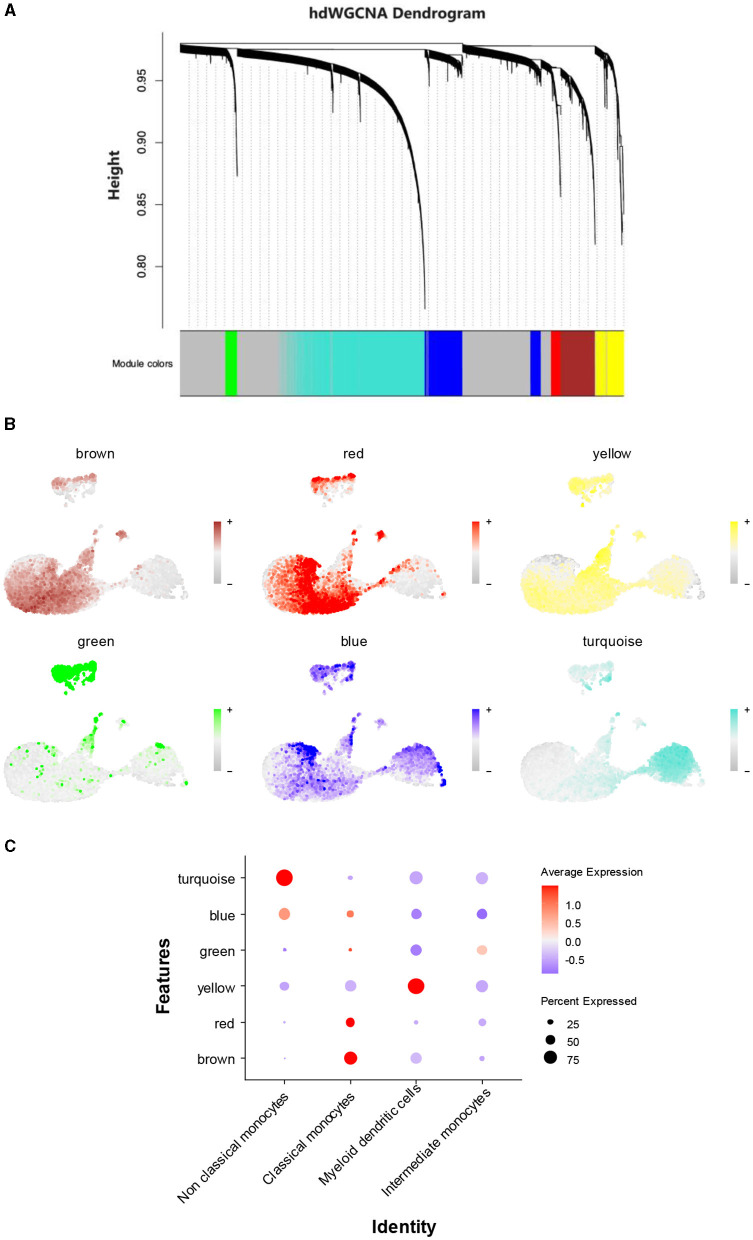
A search for key genetic modules. **(A)** hdWGCNA of non-classical monocytes. **(B)** Visualization of the distribution of each gene module in different monocytes. **(C)** The expression levels of different gene modules in different monocyte cell types.

To identify AD-related gene modules in non-classical monocytes, hdWGCNA was used. All genes were collectively clustered into six non-gray modules. Among these modules, the turquoise and blue modules exhibited the highest expressions (Figure 5A). Visualization of these modular genes revealed that the turquoise module and the blue module were the most widely distributed in the non-classical monocyte region (Figure 5B). Plotting the expression levels of different modules in monocytes illustrated that the turquoise and blue modules were predominantly distributed in non-classical monocytes (Figure 5C). Consequently, 2,262 genes from the turquoise module and 817 genes from the blue module were selected for inclusion in subsequent analyses.

**Figure 6:**
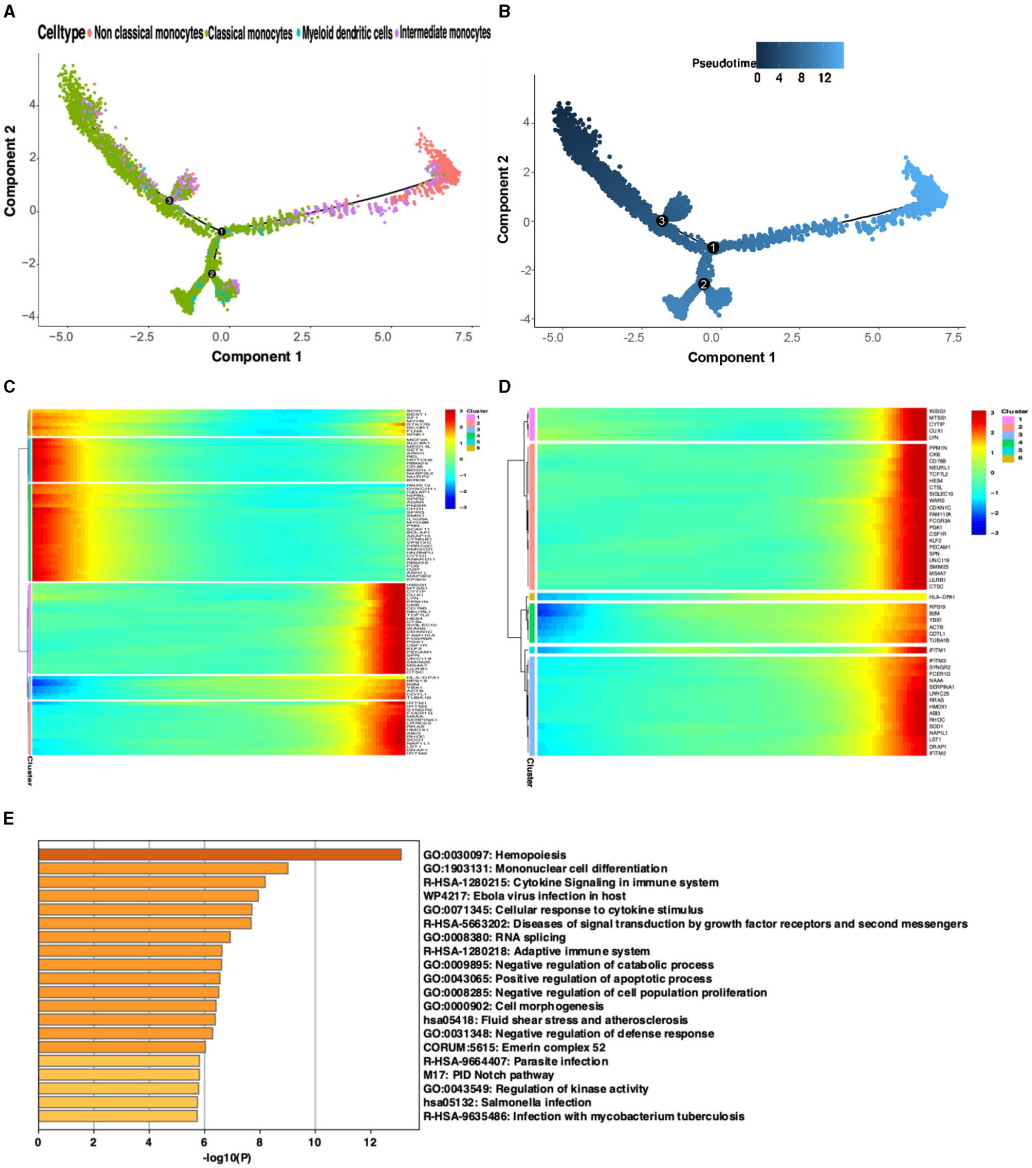
Proposed time-series analysis. **(A)** Indicates different cell clusters; **(B)** Indicates the order of the proposed time from dark to light. **(C)** Visualization of proposed temporal clustering of the blue and turquoise modules. **(D)** Visualization of proposed time-series clustering of the turquoise module. **(E)** Gene enrichment analysis of the top 50 genes in the blue and turquoise modules.

To investigate changes in gene expression during development or transcriptional dynamics in non-classical monocytes, pseudotime analysis was utilized on the integrated dataset to illustrate the on/off status of various genes across pseudotime. The pseudotime analysis revealed that the expression of non-classical monocyte marker genes increased as the pseudotemporal time advanced, indicating a trend for non-classical monocytes to express genes more fully toward the end of the pseudotemporal time compared to other monocytes (Figures 6A, B). To validate this observation, the top 50 differential genes representing non-classical monocytes from the turquoise module and the blue module were clustered into six classes. Visualization of these gene clusters demonstrated that the turquoise module and the blue module were positioned at the end of the pseudotime analysis with more fully expressed genes (Figure 6C). Due to the higher representation of non-classical monocyte genes in the turquoise module, it was selected for further analysis, confirming the same trend as before (Figure 6D). Enrichment analysis of the op 50 genes from the turquoise module and the blue module indicated that these modules were predominantly enriched in immunity, infection, and other related pathways. Their functions were closely associated with hematopoiesis and monocyte differentiation (Figure 6E).

### 3.3 Predictive model construction results for multiple ML algorithms

**Figure 7:**
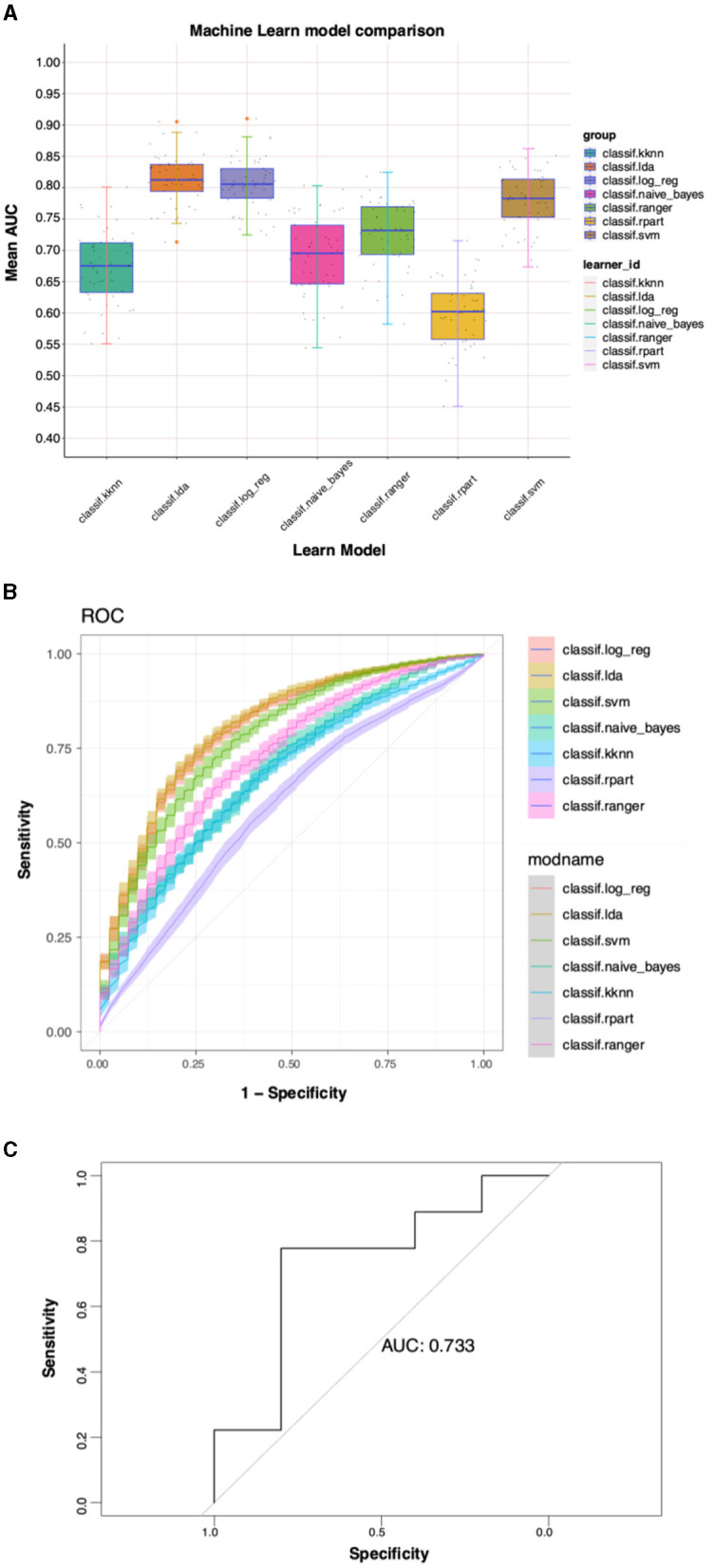
Predictive model construction results for multiple ML algorithms. **(A)** Mean AUC for different ML models. **(B)** Diagnostic ROC curves for different ML models. **(C)** External validation set for ML.

LASSO regression and logistic regression were used to initially screen the previously obtained genes, and 44 genes including “PPM1N”, “CX3CR1”, “WASF2”, “HES4”, “RGS19”, and “CSTB” were obtained and continued to the next step of the analysis. Using the dataset GSE140829 as an internal validation set to verify the accuracy of the machine learning model, the lda and log_reg results show the best test efficacy (AUC = 0.80) (Figures 7A, B). Therefore, the external validation set GSE97760 is selected for lda for the machine learning model, which also shows a good test efficacy (AUC=0.733) (Figure 7C).

### 3.4 MR analysis using predicted genes identified five genes for AD

**Figure 8:**
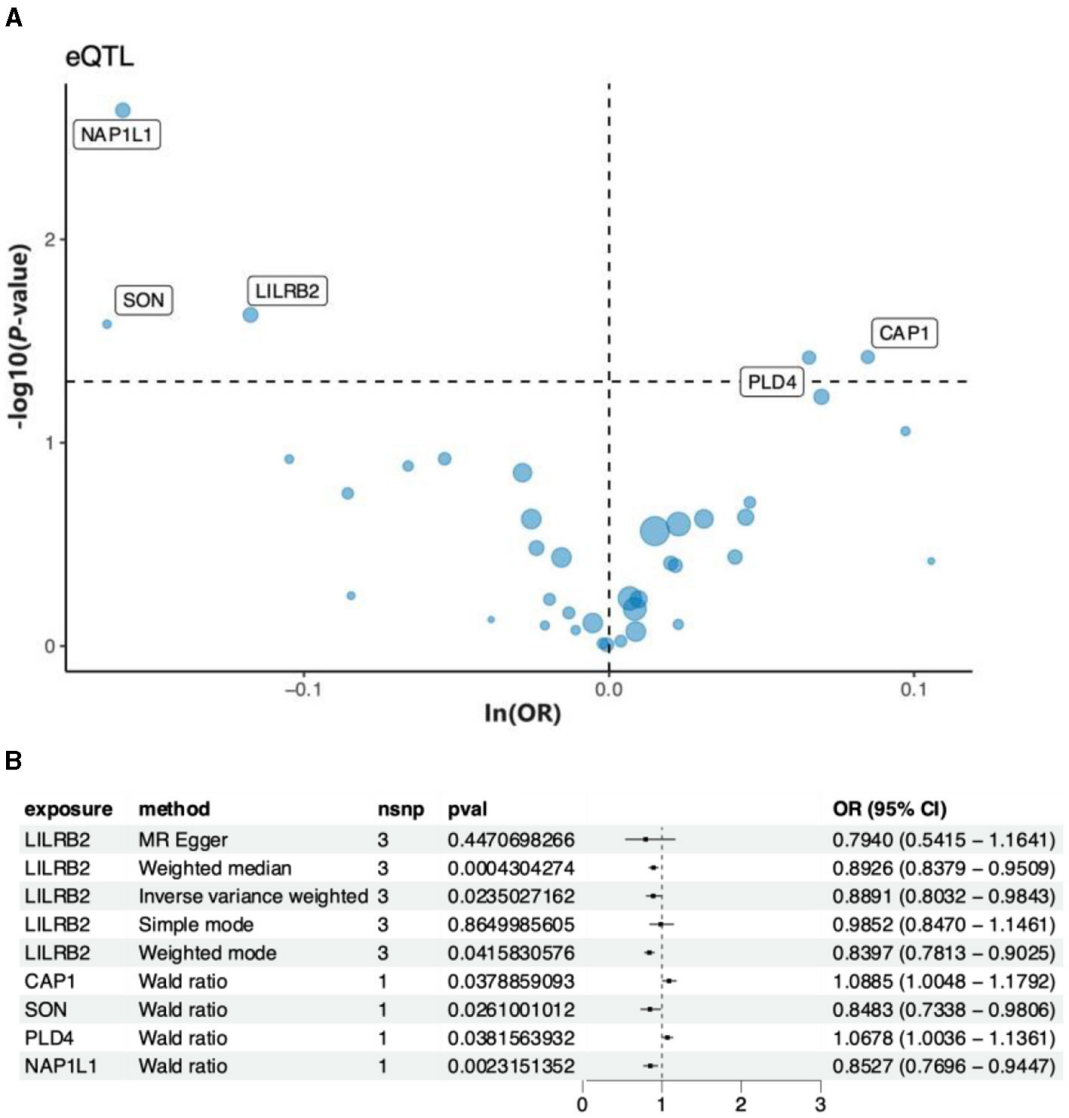
The key genes and AD relationships. **(A)** The volcano map illustrates the relationship between the key genes and AD risk. **(B)** Forest plot depicting the association between key genes and AD risk.

The 44 predicted genes obtained from ML were utilized, and eQTL matching the genes was identified on the GWAS website for MR analysis. This finding aims to identify downstream genes associated with a high risk of AD. Finally, the analysis identified five genes, namely, NAP1L1, SON, L1LRB2, PLD4, and CAP1 that were significantly associated with AD. A volcano plot highlighting genes with significant *p*-values (Figure 8A) was generated to visually illustrate the *p*-value of each gene in relation to the -log10 transformation of ln(OR). The plot clearly displays genes with significant positive and negative correlations. Subsequently, a forest plot was created to visualize the odds ratio (OR) and 95% confidence interval (CI) of each significant gene, emphasizing the robustness and direction of each gene association (Figure 8B). As depicted in the figure, the NAP1L1 gene exhibited a negative correlation with AD. In other words, the risk of AD increased with a decrease in the gene's expression level. Consequently, the NAP1L1 gene was selected for further analysis.

### 3.5 Reverse MR analysis, regional linkage analysis, and the Steiger test of the NAP1L1 genes with AD risk

**Figure 9:**
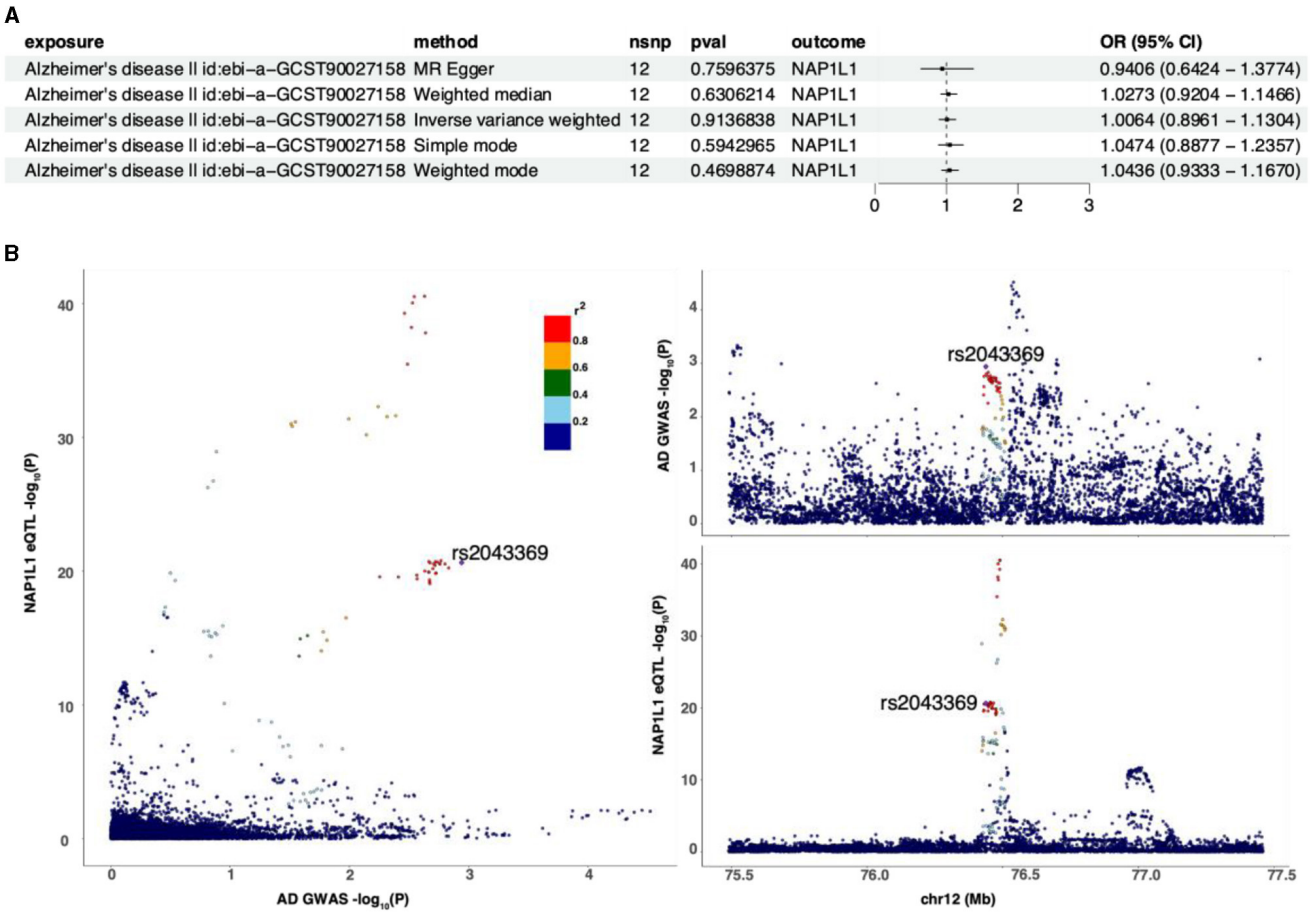
The relationship between the NAP1L1 gene and AD. **(A)** Inverse MR using different MR methods to describe the relationship between AD and the NAP1L1 gene. **(B)** Regional correlation map.

Reverse MR analysis was conducted to assess the causal effect of AD on the NAP1L1 gene, with AD as the exposure and the NAP1L1 gene as the outcome. The analysis revealed no causal relationship between AD and the NAP1L1 gene (OR = 1.0064, 95% CI = 0.8961–1.1304, *p* = 0.9136838 by the IVW method). Additionally, the Steiger test was conducted, and the evaluation between AD and the NAP1L1 genes yielded a TRUE result, signifying the absence of reverse causality (Supplementary Table 1). Consistent results were obtained using MR Egger, weighted median, simple mode, and weighted mode methods (Figure 9A). Initially, regional association plots for the NAP1L1 gene eQTL were displayed in tandem with the AD GWAS results. SNPs indicating a significant association between the NAP1L1 gene and AD were identified through the comparison of the strength of their associations. It can be noted that specific SNP associated with eQTLs for the NAP1L1 gene, such as rs2043359, showed a significant correlation in GWAS for AD. This finding provides preliminary evidence suggesting a potential link between the NAP1L1 gene and AD (Figure 9B). Our findings are generally consistent with our initial hypothesis, suggesting that alterations in the NAP1L1 gene expression modulate the risk of AD.

## 4 Discussion

Single-cell sequencing addresses the limitation of traditional transcriptomics, which only provides an average expression signal for a collection of cells (Du et al., 2023). It can also aid in elucidating the reasons for poor production or clearance of Aβ at the cellular level. Non-classical monocytes travel along the vascular endothelium (Malm et al., 2010; Ong et al., 2018). Intriguingly, these traveling monocytes have been demonstrated to recognize and clear Aβ from the venous lumen of APP/PS1 mice (van de Veerdonk and Netea, 2010). However, the mechanism behind the failure of non-classical monocytes to clear Aβ remains unknown.

We initially verified that non-classical monocytes in AD did not interact with other monocytes as much as in the control group, suggesting that the reduced interactions of non-classical monocytes with other cells have likely led to its reduced clearance of Aβ. This reduction in interactions leads to results that may be related to the TNF-TNFRSF1A signaling pathway. TNFRSF1A, identified as the TNF-alpha receptor, functions as a genetic plasmid that exclusively binds to TNF-alpha. Aβ1-40 induces the activation of several TNF-α-dependent intracellular signaling pathways that play a key role in controlling COX-2 upregulation and activation, synaptic loss, and cognitive decline in mice, which may ultimately lead to AD (Medeiros et al., 2010). Therefore, it is reasonable to assume that a decline in TNF-TNFRSF1A in non-classical monocytes causes a reduction in binding to TNF-α, giving TNF-α the opportunity to be activated by Aβ1-40, causing a series of cascading reactions that ultimately lead to AD. Previous studies and the single-cell sequencing analyses of our study suggest that non-classical monocytes are a key cell population in AD genesis, thus its transcription factors should also be closely related to AD. Furthermore, we conducted literature research on the top-ranked transcription factor, CUX1. Recent studies have shown that the APP intracellular domain (AICD) of the amyloid-β precursor gene is a potential contributor to the development and progression of AD (Konietzko, 2012), and that activation of CUX1 transcriptional activity by the AICD may be implicated in its contribution to AD (Yang et al., 2012). The pseudotime analysis revealed that non-classical monocytes are more likely to exhibit the characteristics of AD cells. Moreover, existing studies have identified cellular senescence in AD (Liu, 2022). Cellular senescence typically occurs at the terminal stage of the cell's growth and development process. We assumed the proliferation of the cell as the starting point and observed the expression of the number of genes in the cell in different states of the cell and found that the expression of non-classical monocytes increased at the end of the pseudotime, in the cellular senescence stage. Enrichment analysis of the turquoise and blue gene modules indicated that these modules are primarily focused on pathways and functions such as hematopoiesis, immunity, monocyte differentiation, infectious disease, and apoptosis. These pathways and functions have also been suggested to be potentially related to AD (Behl, 2000; Feng et al., 2011; Chong et al., 2013; Douros et al., 2021; Chen and Holtzman, 2022).

Because ML is a powerful tool for gene expression analysis, we chose it to screen for genes with high predictive performance (Deo, 2015). To address the limitations of GWAS in fully revealing genetic susceptibility factors for complex diseases, we combined GWAS with eQTL analysis (Zhu et al., 2016; Cano-Gamez and Trynka, 2020). Additionally, we used reverse MR analysis, regional association analysis, and the Steiger test to further validate our findings. Regional association analysis supports the relationship between the NAP1L1 gene and AD. Reverse MR analysis revealed that this causal association did not exist in the reverse direction. Furthermore, the Steiger test confirmed this result. The downregulation of NAP1L1 was found to render cells susceptible to apoptotic cell death by attenuating nuclear factor-κB (NF-κB) transcriptional activity on the anti-apoptotic Mcl-1 gene (Tanaka et al., 2017). Similarly, increased NF-κB expression was found in PBMC samples of AD patients, suggesting that a decrease in the NAP1L1 gene in monocytes might be responsible for AD (Ascolani et al., 2012). NF-κB is a well-recognized inflammatory transcription factor that promotes neurodegeneration and has a huge impact on AD formation (Ju Hwang et al., 2019). In addition, it was experimentally demonstrated that the knockdown of the NAP1L1 gene increased Lys382 acetylation and enhanced the level of pro-apoptotic Bax, thereby promoting cell death (Tanaka et al., 2019). In contrast, Bax is from the Bcl2 family and has pro-apoptotic effects, apoptosis brought about by Bax is thought to be closely related to AD formation (Kumari et al., 2023).

## 5 Conclusion

In summary, our study suggests that the NAP1L1 gene in non-classical monocytes has the potential to serve as a biomarker for predicting AD. However, further functional experiments are required to verify our hypothesis.

## Data availability statement

The original contributions presented in the study are included in the article/supplementary material, further inquiries can be directed to the corresponding author.

## Author contributions

RC: Conceptualization, Writing – original draft, Writing – review & editing, Formal analysis. YX: Data curation, Writing – original draft. ZC: Writing – review & editing. WH: Writing – review & editing. ZH: Conceptualization, Project administration, Supervision, Writing – original draft.
